# Rational treatment options for T1/2N0M0 squamous cell carcinoma of the anal canal: a population-based study combined with external validation

**DOI:** 10.1093/oncolo/oyae068

**Published:** 2024-04-30

**Authors:** Xue Shao, Qiulei Zhang, Ying Huo, Chang Lu

**Affiliations:** Department of Hepatopancreatobiliary Medicine, The Second Hospital of Jilin University, Changchun, People’s Republic of China; Department of Anesthesiology, The Second Hospital of Jilin University, Changchun, People’s Republic of China; Department of Anesthesiology, The Second Hospital of Jilin University, Changchun, People’s Republic of China; Department of Anesthesiology, The Second Hospital of Jilin University, Changchun, People’s Republic of China

**Keywords:** cT1/2N0M0 squamous cell carcinoma of the anal canal, nomogram, prognosis, rational treatment

## Abstract

**Background:**

Treatment options for T1/2N0M0 anal squamous cell carcinoma include chemotherapy, radiotherapy, chemoradiotherapy, and local excision, although the optimal treatment method has not been determined.

**Methods:**

The National Cancer Institute Surveillance, Epidemiology and Results database was used to search and screen 1465 patients with cT1/2N0M0 anal squamous cell carcinoma who were clinically diagnosed between 2004 and 2016. Survival analysis was performed using the Kaplan-Meier method and log-rank test. Cox proportional hazards regression analysis was performed to screen independent prognostic factors and build a nomogram survival prediction model. According to the risk score, patients were divided into low, medium, and high risk groups using X-tile software.

**Results:**

Age, sex, grade and cT stage were identified as independent prognostic factors for cT1/2N0M0 anal squamous cell carcinoma and were included in the nomogram to construct a prediction model. The C-index of the model was 0.770 [95% confidence interval (CI), 0.693-0.856], which was higher than the C-index of T stage 0.565 (95% CI, 0.550-0.612). Low-risk patients benefited from local resection, moderate-risk patients benefited from radiotherapy, and high-risk patients benefited from radiotherapy or chemoradiotherapy. This was confirmed using external validation data from the center.

**Conclusion:**

The nomogram developed in this study effectively and comprehensively evaluated the prognosis of patients with cT1/2N0M0 squamous cell carcinoma of the anal canal. Local excision is recommended for low risk patients, radiotherapy for moderate-risk patients, and radiotherapy or chemoradiotherapy for high-risk patients.

## Teaser

Considering the treatment options for patients with T1/2N0M0 anal squamous cell carcinoma, this article describes a nomogram to stratify patients by incorporating high risk factors, which is essential for providing individualized guidance regarding the comprehensive treatment of patients.

**Implications for Practice**

Because the treatment of T1/2N0M0 anal squamous cell carcinoma is controversial, the authors developed a nomogram to stratify patients by incorporating high-risk factors, which is essential for providing individualized guidance regarding the comprehensive treatment of patients.

## Background

The 2020 Global Cancer Statistics reported 50 865 new cases of anal canal cancer and 19 293 deaths.^1^ Although the incidence of anal canal cancer is lower than that of colorectal cancer, it is increasing yearly and it is more common in women, underscoring the need to pay attention to this disease.^2^ Squamous cell carcinoma of the anus (SCCA) is the most common anal canal cancer, and its incidence is related to high-risk factors such as HIV, HPV, smoking, history of sexually transmitted diseases, cervical cancer, perineal tumors, and immunosuppression.^3-5^ The treatment of SCCA has changed from combined abdominal and perineal resection in the 1970s to a comprehensive treatment based on radiotherapy (RT) and chemotherapy (CT). The advantage of this treatment is that it can preserve the anus and improve the quality-of-life of patients. For limited stage patients, it can achieve a 70%-90% local control rate and a 60%-70% 5-year disease-free survival rate. The curative effect is similar to that of surgical treatment and can be more effective than simple surgery.^6-8^ Surgery is used as a salvage treatment for most stages I-III SCCAs or as a major treatment for early perianal skin cancer.

Although chemoradiotherapy (CRT) preserves the patient’s anus, anal tone usually decreases after the completion of CRT, and sexual function is also affected. For SCCA located at the anal verge and tumors < 2 cm, local excision (LE) may be more effective.^9^ Leona et al found that LE alone resulted in higher local recurrence rates and poorer prognosis in T1-2N0M0 patients.^10^ A study in 1996 reported that CRT increases CSS and decreases local recurrence compared with RT alone, whereas it has no effect on overall survival (OS).^11^ Although current guidelines recommend concurrent CRT for SCCA, previous studies either did not include stage I or included a low proportion of T1/2N0M0 patients.^12,13^ Therefore, reasonable treatment methods for early SCCA patients need to be identified.

In this study, we evaluated the prognosis of patients with T1/2N0M0 SCCA using clinical case factor analysis in the Surveillance, Epidemiology, and End Results (SEER) database. Patients were divided into low-, moderate-, and high-risk groups according to the nomogram score, and those who benefited from LE, CT, RT, and CRT were selected.

## Methods

### Patient cohort

SEER*Stat (version 8.4.0) software was used to identify 1465 patients with cT1/2N0M0 SCCA diagnosed between 2004 and 2016. The inclusion criteria were as follows: (1) pathologically diagnosed patients with anal canal cancer (ICD-O-3: C21.0-C21.2), (2) complete follow-up and survival data, (3) not receiving neoadjuvant radiotherapy, (4) pathology was squamous cell carcinoma, and (5) primary anal canal cancer. The variables analyzed in this study and survival information. Further exclusions were made if information on the above variables was unknown.

### Statistical analysis

Clinical and pathological factors were analyzed. Establish a prediction model: (1) univariate and multivariate COX analyses were performed to determine the correlation between each variable and OS; (2) according to the results of multivariate analysis, variables with *P* < .05 were included and a nomogram survival prediction model was established. The performance of the prediction model was tested. Discrimination was measured by the C-index,^14^ and the C-index of the nomogram and T stage was compared to evaluate the clinical performance of the model. The calibration curve was used to evaluate the consistency between the predicted survival rate and the actual survival rate. Decision curve analysis (DCA) was used to evaluate the clinical net benefit and compare it with T stage. (3) X-tile software was used to divide cases into 3 groups according to the risk score of the nomogram.^15^ All statistical analyses in this study were performed using SPSS 24.0 and R language (version 4.0.0), and *P* < .05 was considered statistically significant.

## Results

### Patient demographics

**Figure 1.**
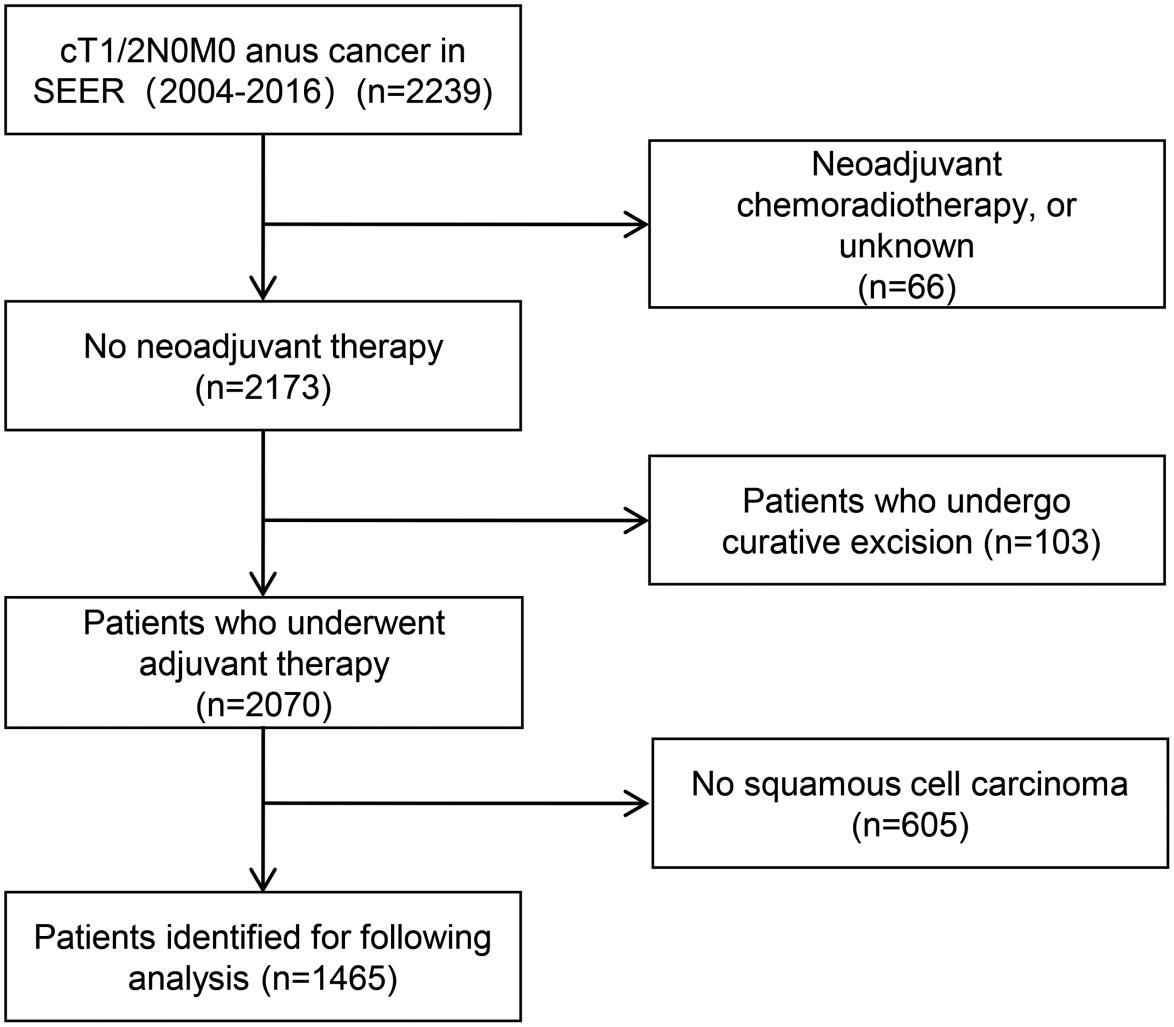
Flowchart of the selection process of included patients.

**Figure 2.**
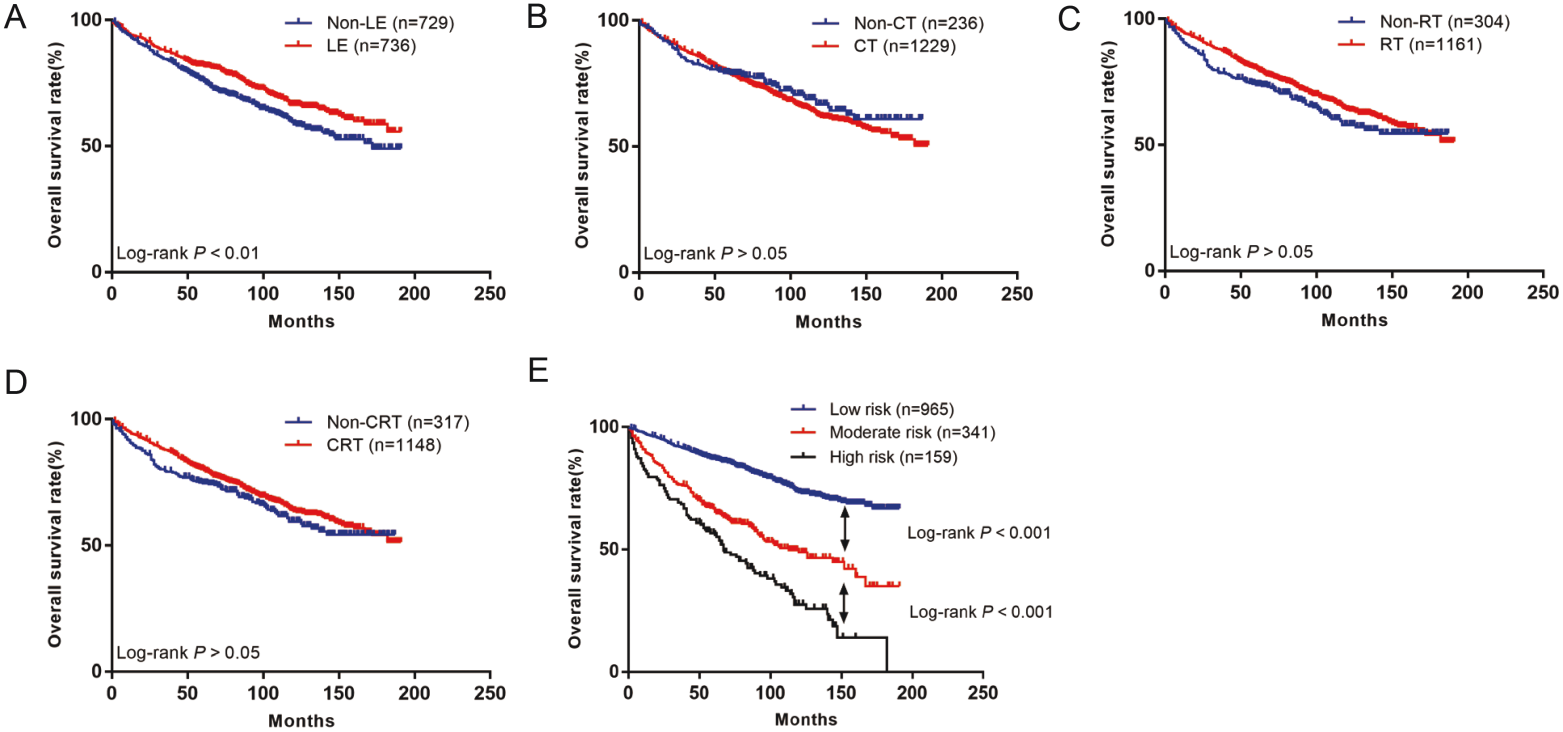
The Kaplan-Meier curves of OS for patients in our study. (A) OS for patients with LE, (B) OS for patients with CT, (C) OS for patients with RT, (D) OS for patients with CRT, (E) OS in different subgroups of all patients.

After excluding confounding factors, a total of 1465 patients with cT1/2N0M0 SCCA were finally included (Figure 1), the distribution of pathological factors of patients is shown in Table 1. Survival curves showed that LE surgery had a better prognosis than non-LE (Figure 2A); CT (Figure 2B), RT (Figure 2C), and CRT (Figure 2D) did not improve patient outcomes.

**Table 1.** Characteristics of patients.

Variable	Total [*n* (%)]
Age	
≤ 70	1177(80.3)
> 70	288(19.7)
Sex	
Male	580(39.6)
Female	885(60.4)
Race	
White	1270(86.7)
Black	132(9.0)
API	46(3.1)
Other	17(1.2)
Grade	
Well/moderately	715(48.8)
Poorly/undifferentiated	372(25.4)
Unknown	378(25.8)
Size (cm)	
< 1	139(9.5)
≥ 1	1326(90.5)
T stage	
T1	644(44.0)
T2	821(56.0)
Interval time	
0 month	678(46.3)
≥ 1 month	787(53.7)

Abbreviation: API, Asian/Pacific Islander.

### Construction of the nomogram

**Figure 3.**
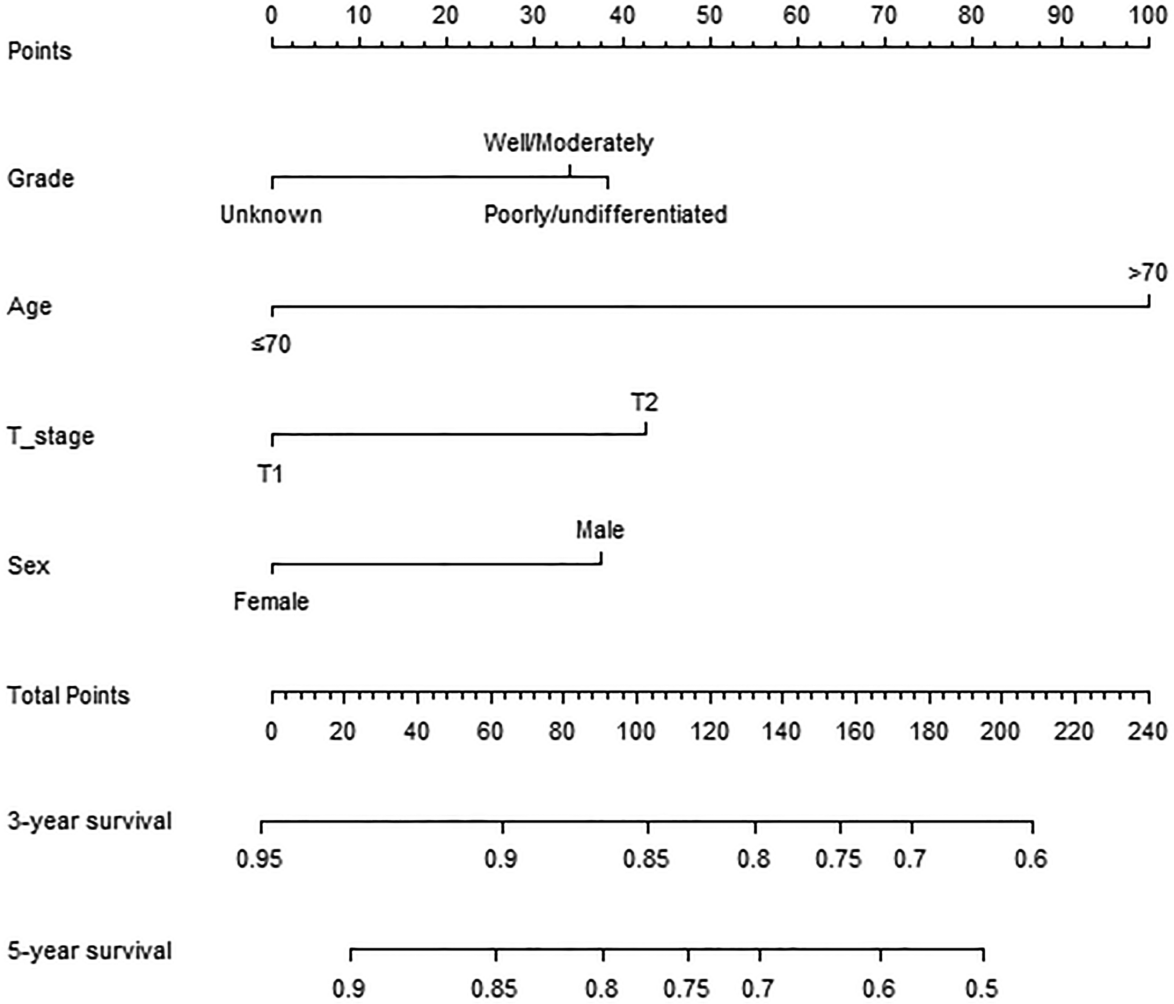
Oncologic nomogram for T1/2N0M0 SCCA.

The pathological factors of all patients were included in the COX hazard proportional model, which showed that age, sex, grade, and T stage were independent prognostic factors for patients with SCCA (*P* < .05; Supplementary Table S1). On this basis, a nomogram was constructed to predict the 3- and 5-year survival rates of T1/2N0M0 SCCA (Figure 3).

### Effectiveness of the prediction model

**Figure 4.**
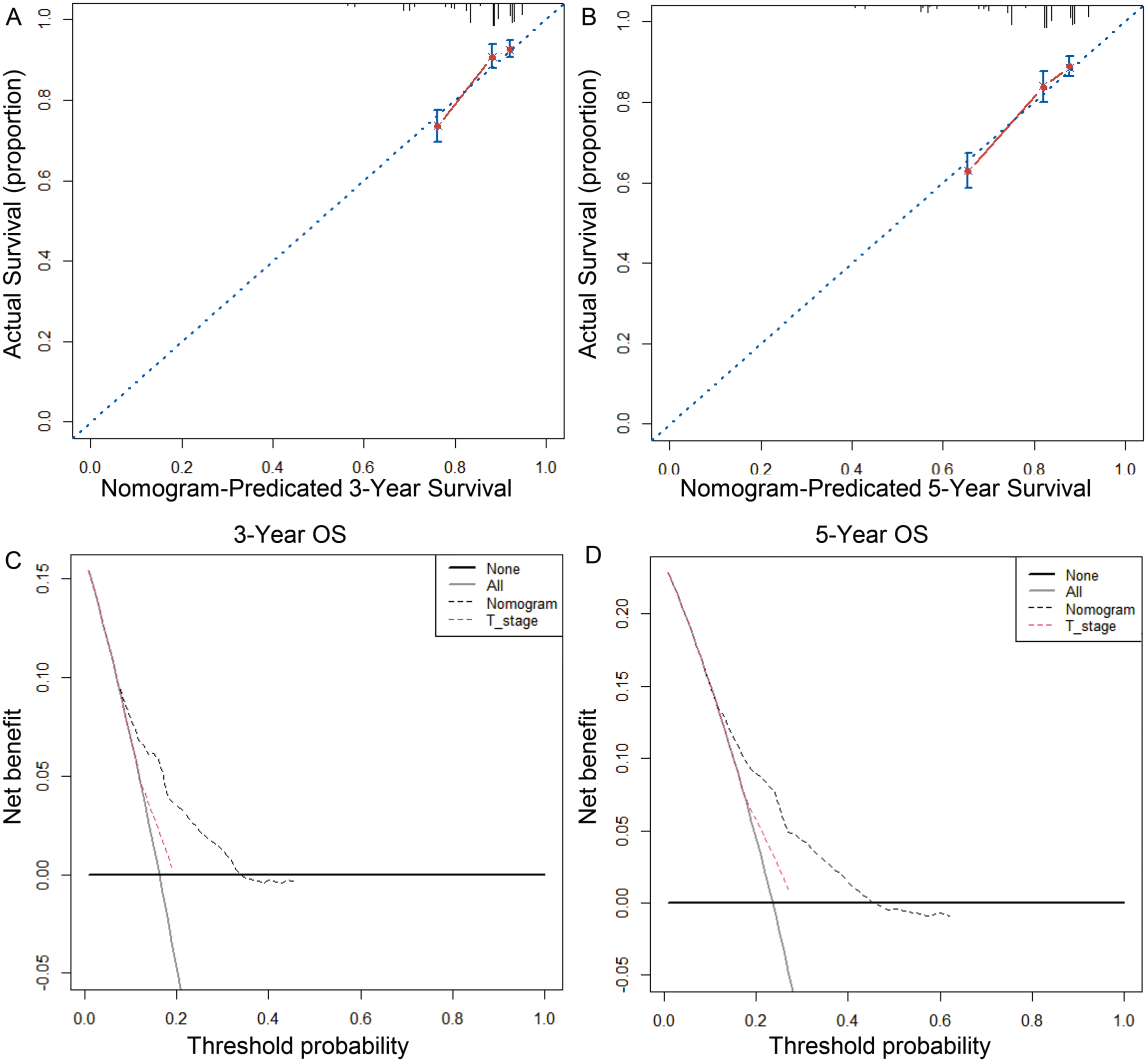
Calibration curves and decision curve for OS prediction: (A) 3-year OS calibration curve in our cohort; (B) 5-year OS calibration curve in our cohort; (C) Nomogram were compared to the T stage in terms of 3-year OS in our decision curve analysis; (D) Nomogram were compared to the T stage in terms of 5-year OS in our decision curve analysis.

The C-index of the nomogram incorporating the above 4 risk factors to predict prognosis was 0.770 [95% confidence interval (CI), 0.693-0.856], which was significantly higher than the C-index of T stage at 0.565 (95% CI, 0.550-0.612) for predicting prognosis. The nomogram calibration curves of 3- and 5-year OS showed that the predicted survival probability was in good agreement with the actual survival probability (Figure 4A, B). The DCA determined that the net income of the nomogram prognosis model for different decision thresholds was higher than the prediction line of the T stage system (Figure 4C, D).

### Risk stratification system for overall patients

**Figure 5.**
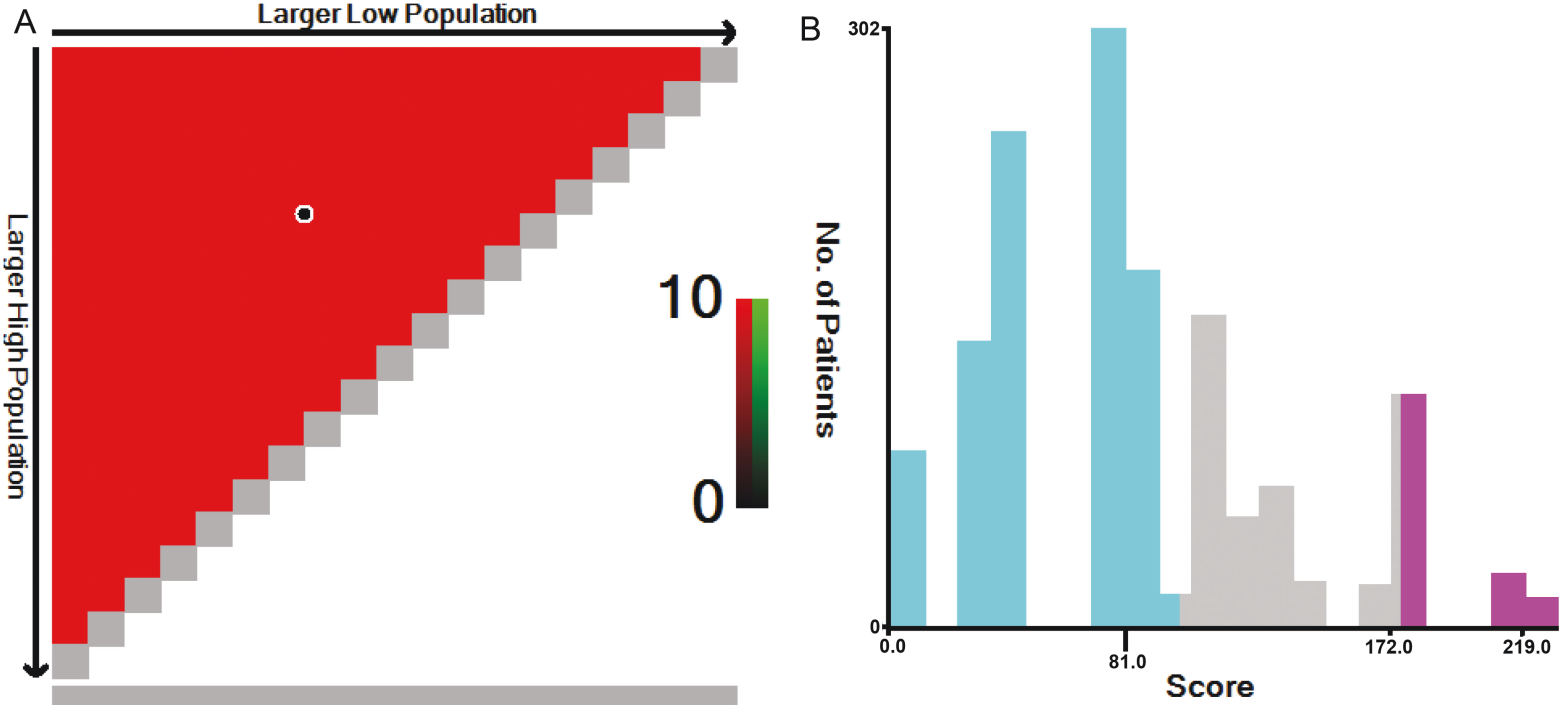
X-tile analysis for risk stratification: (A) The optimal cutoff value, (B) numbers of patients in low-, moderate-, and high-risk subgroups.

The score of each patient was calculated using the nomogram (Supplementary Table S2), and patients were divided into 3 risk groups based on 2 cutoff values determined using X-tile software (Figure 5A, B): low-risk group (score ≤ 81, *n* = 965), moderate-risk group (score 100-172, *n* = 341), and high-risk group (score ≥ 176, *n* = 159). The 5-year survival rates of the low-, moderate-, and high-risk groups were 87.1%, 66.8%, and 56.8% respectively, and the differences were statistically significant (*P* < .05, Figure 2E).

### Efficacy of LE for patients in different groups

**Figure 6.**
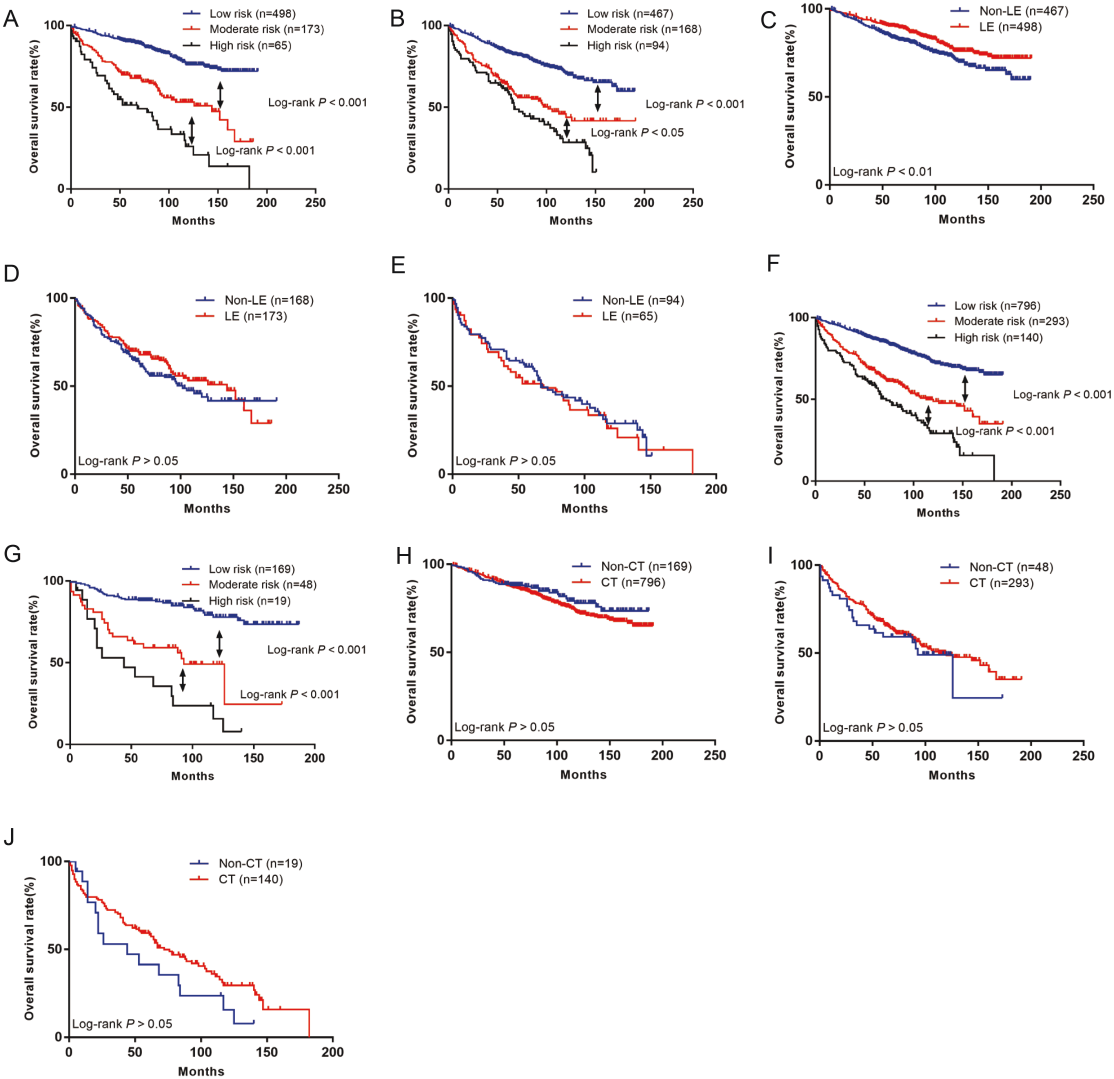
The Kaplan-Meier curves of OS for patients. (A) OS in different risk subgroups of non-LE group, (B) OS in different risk subgroups of LE group, (C) OS for patients with or without LE in low-risk group, (D) OS for patients with or without LE in moderate-risk group, (E) OS for patients with or without LE in high-risk group, (F) OS in different risk subgroups of non-CT group, (G) OS in different risk subgroups of CT group, (H) OS for patients with or without CT in low-risk group, (I) OS for patients with or without CT in moderate-risk group, (J) OS for patients with or without CT in high risk group.

Based on the existing scoring system, we divided the non-LE group into 3 groups. The 5-year survival rates of the 3 groups were 90.2%, 69.3%, and 51.4%, respectively, and the difference was statistically significant (*P* < .05, Figure 6A). In the LE group, the 5-year survival rates of the low, moderate and high risk groups were 83.6%, 64.2%, and 60.4%, respectively, and the difference was statistically significant (*P* < .05, Figure 6B). The results showed that patients in the low-risk group benefited from LE (Figure 6C), whereas patients in the moderate-risk group (Figure 6D) and those in the high-risk group did not benefit from LE (Figure 6E).

### Efficacy of CT for patients in different groups

We divided the non-CT group into 3 groups. The 5-year survival rates of the 3 groups were 86.8%, 68.1%, and 58.7%, respectively, and the difference was statistically significant (*P* < .05, Figure 6F). In the CT group, the 5-year survival rates of the low-, moderate-, and high-risk groups were 88.1%, 59.3%, and 41.3%, respectively, and the difference was statistically significant (*P* < .05, Figure 6G). Assessment of the benefit of CT in the different groups showed that patients in the low-risk group (Figure 6H), patients in the moderate-risk group (Figure 6I), and patients in the high-risk group did not benefit from CT (Figure 6J).

### Efficacy of RT for patients in different groups

We divided the non-RT group into 3 groups: low, moderate, and high. The 5-year survival rates of the 3 groups were 87.1%, 69.5% and 63.2%, respectively, and the difference was statistically significant (*P* < .05, Supplementary Figure S1A). In the RT group, the 5-year survival rates of the low-, moderate-, and high-risk groups were 86.8%, 55.8%, and 35.8%, respectively, and the difference was statistically significant (*P* < .05, Supplementary Figure S1B). The results showed that patients in the low-risk group did not benefit from RT (Supplementary Figure S1C), whereas patients in the moderate-risk group (Supplementary Figure S1D) and patients in the high-risk group benefited from RT (Supplementary Figure S1E).

### Efficacy of CRT for patients in different groups

We divided the non-CRT group into 3 groups: low, moderate, and high. The 5-year survival rates of the 3 groups were 87.0%, 69.3%, and 62.9%, respectively, and the difference was statistically significant (*P* < .05, Supplementary Figure S1F). In the CRT group, the 5-year survival rates of the low-, moderate-, and high-risk groups was statistically significant (*P* < .05, Supplementary Figure S1G). Assessment of the benefit of CRT in the different groups showed that patients in the low-risk group (Supplementary Figure S1H) and patients in the moderate-risk group did not benefit from CRT (Supplementary Figure S1I), whereas patients in the high-risk group benefited from CRT (Supplementary Figure S1J).

### Comprehensive treatment for patients in the external validation group

According to the existing scoring system, patients in the external validation group were divided into low-risk (score ≤ 81, *n* = 114), moderate-risk (score 100-172, *n* = 133), and high-risk groups (score ≥ 176, *n* = 46). The 5-year survival rates of the low-, moderate-, and high-risk groups was not statistically significant (*P* > .05, Supplementary Figure S2A).

We divided the non-LE group into 3 groups: low, moderate, and high. The 5-year survival rates of the 3 groups were 86.0%, 72.5% and 74.0%, respectively, and the difference was not statistically significant (*P* > .05, Supplementary Figure S2B). In the LE group, the 5-year survival rates of the low-, moderate-, and high-risk groups was not statistically significant (Supplementary Figure S2C). Analysis of the benefit of LE in the different groups showed that patients in the low-risk group benefited from LE (Supplementary Figure S2D), whereas patients in the moderate-risk group (Supplementary Figure S2E) and patients in the high-risk group did not benefit from LE (Supplementary Figure S2F).

We divided the non-CT group into 3 groups, the 5-year survival rates of the 3 groups were 85.8%, 71.9%, and 74.7%, respectively, and the difference was not statistically significant (*P* > .05, Supplementary Figure S2G). In the CT group, the 5-year survival rates of the low-, moderate-, and high-risk groups were 100.0%, 90.3%, and 50.0%, respectively, and the difference was not statistically significant (*P* > 0.05, Supplementary Figure S2H). Assessment of the benefit of CT in the different groups showed that patients in the low-risk group (Supplementary Figure S2I), patients in the moderate-risk group (Supplementary Figure S2J), and patients in the high-risk group did not benefit from CT (Supplementary Figure S2K).

We divided the non-RT group into 3 groups, the 5-year survival rates of the 3 groups were 86.9%, 70.0%, and 64.4%, respectively, and the difference was statistically significant (*P* < .05, Supplementary Figure S3A). In the RT group, the 5-year survival rates of the low-, moderate-, and high-risk groups were 100.0%, 87.5%, and 92.9%, respectively, and the difference was not statistically significant (*P* > .05, Supplementary Figure S3B). Assessment of the benefit of RT in the different groups showed that patients in the low-risk group did not benefit from RT (*P* > 0.05, Supplementary Figure S3C), whereas patients in the moderate-risk group (Supplementary Figure S3D) and patients in the high-risk group benefited from RT (Supplementary Figure S3E).

We divided the non-CRT group into 3 groups, the 5-year survival rates of the 3 groups were not statistically significant (*P* > 0.05, Supplementary Figure S3F). In the CRT group, the 5-year survival rates of the low, moderate, and high risk groups were 100.0%, 100.0% and 50.0%, respectively, and the difference was not statistically significant (*P* > .05, Supplementary Figure S3G). Assessment of the benefit of CRT in the different groups showed that patients in the low-risk group (*P* > .05, Supplementary Figure S3H), patients in the moderate-risk group (*P* > .05, Supplementary Figure S3I), and patients in the high-risk group did not benefit from CRT (Supplementary Figure S3J).

## Discussion

Before CRT became the treatment standard for SCCA, abdominoperineal combination surgery was the main treatment method. Although the OS rate of patients treated with abdominoperineal combination surgery was approximately 70%, it led to permanent colostomy and was associated with several inconveniences in patients’ lives.^16^ As the remission rate of CRT reached 84%, CRT became the gold standard in the treatment of SCCA.^17^ However, many studies did not include stage I and T2N0M0 patients. For example, the RTOG 98-11 study showed that CRT improved OS and DFS, but the study did not include stage I patients.^18^ Although some RCT studies achieved good results of CRT based on 5-Fu, these studies did not include T1/2N0M0 SCCA patients.^11,19-21^ Bosset et al found that the local recurrence rate of T1N0M0 SCCA patients receiving CRT was lower than that of patients treated with CT alone (RR = 0.35; 95% CI: 0.12-0.97; *P* < .05).^22^ Although all T1/2N0M0 SCCA patients need CRT, some patients can be cured by LE. Whether CRT will lead to excessive treatment is a concern of our study.

This study provided guidelines for selecting the optimal treatment for T1/2N0M0 SCCA by analyzing the prognostic factors of patients. Leon et al^10^ found that old age was not a high-risk factor for RFS (*P* = .09), whereas in this study, older patients had a worse prognosis (HR = 2.960; 95% CI: 2.443-3.588; *P* < .001). This result could be related to the presence of basic diseases in the elderly, decreased sensitivity and tolerance to CRT, and a higher incidence of adverse reactions. Although SCCA is more common among women, Leon et al^10^ reported that female sex was not a high-risk factor affecting RFS (*P* = .51). The results of the present study showed that women had a better prognosis than men (*P* < .001), which may be related to the higher clinical benefit rate in women receiving CRT in this study (73.3%). T staging of SCCA is based on tumor size as follows: T1 ≤ T1 cm; 2 < T2 ≤ 5 cm. A higher staging indicates larger tumors and thus a worse prognosis (*P* < .001). The nomogram developed in this study included sufficient risk factors and effectively predicted the 3- and 5-year survival rates of T1/2N0M0 SCCA.

There are no high-quality studies demonstrating that surgery or CRT is the best choice for patients with T1/2N0M0 SCCA.^8,23^ Certain benefit from LE treatment, mainly those with small lesions (2 cm) located in the anal margin skin. Cases in which a safe margin >5 mm can be obtained while preserving anal sphincter function may benefit from LE. In a large study involving 2243 patients between 2004 and 2012, 503 patients received LE and 1740 received CRT. Since 2004, the proportion of patients undergoing LE has increased yearly, and most of the patients with tumors measuring approximately 1 cm undergo LE. The survival analysis showed that the 5-year OS of patients who underwent LE was 85.3% vs. that of CRT at 86.8% (*P* = .93).^13^ This study found that LE has no disadvantage compared with CRT, and the advantages of LE include lower hospitalization costs and fewer complications among others. In the SCCA guideline, few or no T1/2N0M0 patients were included in studies of CRT for patients with SCCA.^18-21^ Therefore, the treatment of early SCCA remains controversial. Many physicians believe that CRT is an overtreatment for early patients; it is important to select patients who will benefit from or require CRT.^9,24^ We found that low-risk patients who underwent LE gained a survival benefit (*P* < .01), whereas those who received CT, RT, and CRT did not show a survival benefit. Moderate-risk patients benefited from RT (*P* = .03) but did not benefit from LE, CT, and CRT. High-risk patients benefited from RT (*P* < .01) and CRT (*P* < .01), while patients did not benefit from LE and CT. External validation data indicated that high risk patients benefited from RT but not from CRT. Therefore, we recommend that high risk patients receive RT alone. Despite the long-term toxicity of CRT, the present study did not assess long-term quality of life, which is why we recommend RT for high-risk patients to reduce the long-term toxicity of CT.^25^ Buckstein et al^26^ showed that in elderly patients with stage I SCCA, there are no statistically significantly differences between CRT and RT alone regarding OS (HR = 0.7; 95% CI: 0.4-1.0; *P* > .05), CSS (HR = 0.7; 95% CI: 0.3-1.6; *P* > .05), colorectal free survival (HR = 1.1; 95% CI: 0.5-2.5; *P* > .05), and DFS (HR = 0.9; 95% CI: 0.6-1.4; *P* > .05), which is consistent with the present data.

After RT, patients with SCCA can experience skin molting, diarrhea, pain, and dysuria. These symptoms are aggravated after CT with 5-Fu and mitomycin. In addition, malignant complications such as hemorrhagic enteritis in the late stage are 2-fold more common than with RT alone. The phase III clinical trial ECOG EA2182 therefore recommend customizing RT for T1/2N0M0 SCCA,^27^ we are also looking forward to the ongoing experimental results (Anal Cancer Trial 4). There is currently no independent CT study, and the present validation did not demonstrate that CT alone is effective, which may be related to the insensitivity of SCCA.^28^ However, studies show that the pathological complete response rate for cetuximab plus CRT is 95%, the median survival period is 43.4 months, and the 3-year local control rate is 64.2%.^29^ Although CRT can cure patients with SCCA, more and more clinical experiments have taken into account the complications of treatment and the risk factors of recurrence.^30^ SCCA has high requirements on the accuracy of clinical staging, and many factors affect its accurate staging, such as peripheral lymph node metastasis can only be determined by imaging, whether lymph node biopsy in the groin area is done or not, accuracy of imaging, etc. Due to the change of tumor staging, its prognosis and treatment plan will change significantly. Therefore, it is still a long way to promote the standardized treatment of SCCA, and the exploration of new diagnosis and treatment methods should not be ignored.

The present study had several limitations. Because of the lack of data in the database, HPV infection and HIV positivity, common risk factors for anal cancer patients, was not included in the analysis. The inconsistency of CT regimen and RT dose will increase the bias of our article. Patients’ underlying health conditions, such as hepatitis C, can also contribute to bias. The present study was a retrospective study, and some patients were not included because of data loss, which may lead to bias. At present, the treatment of T1/2N0M0 SCCA is controversial. We developed a model to stratify patients by incorporating high risk factors, which is essential for providing individualized guidance regarding the comprehensive treatment of patients, underscoring the importance of this study.

## Conclusion

Stratification of patients using the proposed model led to the recommendation of RT for moderate and high-risk patients and LE for low-risk patients.

## Supplementary Material

oyae068_suppl_Supplementary_Table_S1

oyae068_suppl_Supplementary_Table_S2

oyae068_suppl_Supplementary_Figures_S1-S3

## Data Availability

The data underlying this article will be shared on reasonable request to the corresponding author.
