# Supplementary material for: Rational treatment options for T1/2N0M0 squamous cell carcinoma of the anal canal: a population-based study combined with external validation
Source: Oncologist. 2024 Apr 30;29(8):e1003–11. doi: 10.1093/oncolo/oyae068 (PMC11299955; doi:10.1093/oncolo/oyae068)
Supplement: oyae068_suppl_Supplementary_Table_S1 [file oyae068_suppl_supplementary_table_s1.docx]

Table S1 The univariate and multivariate analyses of factors associated with overall survival

| Variable | univariate Cox regression | | multivariate Cox regression | |
| --- | --- | --- | --- | --- |
|  | HR (95% CI) | P-value | HR (95% CI) | P-value |
| Age |  |  |  |  |
| ≤70 | 1 |  | 1 |  |
| >70 | 2.975 (2.459-3.599) | <0.001 | 2.960(2.443-3.588) | <0.001 |
| Sex |  |  |  |  |
| Male | 1 |  | 1 |  |
| Female | 0.745(0.621-0.893) | 0.001 | 0.665(0.552-0.800) | <0.001 |
| Race |  |  |  |  |
| White | 1 |  |  |  |
| Black | 1.281(0.959-1.710) | 0.093 |  |  |
| API | 1.240(0.773-1.990) | 0.371 |  |  |
| Other | 0.956(0.357-2.561) | 0.929 |  |  |
| Grade |  |  |  |  |
| Well/moderately | 1 |  | 1 |  |
| Poorly/undifferentiated | 1.056(0.856-1.303) | 0.610 | 1.048(0.846-1.298) | 0.668 |
| Unknown | 0.657(0.516-0.835) | 0.001 | 0.691(0.543-0.879) | 0.003 |
| Size (cm) |  |  |  |  |
| <1 | 1 |  |  |  |
| ≥1 | 1.541(1.076-2.206) | 0.018 |  |  |
| T stage |  |  |  |  |
| T1 | 1 |  | 1 |  |
| T2 | 1.703(1.407-2.061) | <0.001 | 1.590(1.312-1.972) | <0.001 |
| Interval time |  |  |  |  |
| 0 month | 1 |  |  |  |
| ≥1 month | 1.153(0.961-1.384) | 0.126 |  |  |

API, Asian/Pacific Islander
