# Supplementary material for: Rational treatment options for T1/2N0M0 squamous cell carcinoma of the anal canal: a population-based study combined with external validation
Source: Oncologist. 2024 Apr 30;29(8):e1003–11. doi: 10.1093/oncolo/oyae068 (PMC11299955; doi:10.1093/oncolo/oyae068)
Supplement: oyae068_suppl_Supplementary_Table_S2 [file oyae068_suppl_supplementary_table_s2.docx]

Table S2 Point assignment of each component and prognostic score for SCCA

| Group | Score | Estimated 3-y OS (%) | Estimated 5-y OS (%) |
| --- | --- | --- | --- |
| Age |  |  |  |
| ≤70 | 0 |  |  |
| >70 | 100 |  |  |
| Sex |  |  |  |
| Male | 38 |  |  |
| Female | 0 |  |  |
| Grade |  |  |  |
| Well/moderately | 34 |  |  |
| Poorly/undifferentiated | 38 |  |  |
| Unknown | 0 |  |  |
| T stage |  |  |  |
| T1 | 0 |  |  |
| T2 | 43 |  |  |
| Total score |  |  |  |
|  | -3 | 95 |  |
|  | 63 | 90 |  |
|  | 103 | 85 |  |
|  | 132 | 80 |  |
|  | 156 | 75 |  |
|  | 175 | 70 |  |
|  | 208 | 60 |  |
|  | 22 |  | 90 |
|  | 62 |  | 85 |
|  | 91 |  | 80 |
|  | 114 |  | 75 |
|  | 134 |  | 70 |
|  | 167 |  | 60 |
