# Supplementary material for: Rational treatment options for T1/2N0M0 squamous cell carcinoma of the anal canal: a population-based study combined with external validation
Source: Oncologist. 2024 Apr 30;29(8):e1003–11. doi: 10.1093/oncolo/oyae068 (PMC11299955; doi:10.1093/oncolo/oyae068)
Supplement: oyae068_suppl_Supplementary_Figures_S1-S3 [file oyae068_suppl_supplementary_figures_s1-s3.zip › Figure S1-S3-Captions.docx]

Figure S1 The Kaplan-Meier curves of OS for patients. (A) OS in different risk subgroups of non-RT group; (B) OS in different risk subgroups of RT group; (C) OS for patients with or without RT in low risk group; (D) OS for patients with or without RT in moderate risk group; (E) OS for patients with or without RT in high risk group; (F) OS in different risk subgroups of non-CRT group; (G) OS in different risk subgroups of CRT group; (H) OS for patients with or without CRT in low risk group; (I) OS for patients with or without CRT in moderate risk group; (J) OS for patients with or without CRT in high risk group

Figure S2 The Kaplan-Meier curves of OS for patients in external validation group. (A) OS in different risk subgroups of all patinests; (B) OS in different risk subgroups of non-LE group; (C) OS in different risk subgroups of LE group; (D) OS for patients with or without LE in low risk group; (E) OS for patients with or without LE in moderate risk group; (F) OS for patients with or without LE in high risk group; (G) OS in different risk subgroups of non-CT group; (H) OS in different risk subgroups of CT group; (I) OS for patients with or without CT in low risk group; (J) OS for patients with or without CT in moderate risk group; (K) OS for patients with or without CT in high risk group

Figure S3 The Kaplan-Meier curves of OS for patients in external validation group. (A) OS in different risk subgroups of non-RT group; (B) OS in different risk subgroups of RT group; (C) OS for patients with or without RT in low risk group; (D) OS for patients with or without RT in moderate risk group; (E) OS for patients with or without RT in high risk group; (F) OS in different risk subgroups of non-CRT group; (G) OS in different risk subgroups of CRT group; (H) OS for patients with or without CRT in low risk group; (I) OS for patients with or without CRT in moderate risk group; (J) OS for patients with or without CRT in high risk group
