# Supplementary figures and images for: Rational treatment options for T1/2N0M0 squamous cell carcinoma of the anal canal: a population-based study combined with external validation
Source: Oncologist. 2024 Apr 30;29(8):e1003–11. doi: 10.1093/oncolo/oyae068 (PMC11299955; doi:10.1093/oncolo/oyae068)

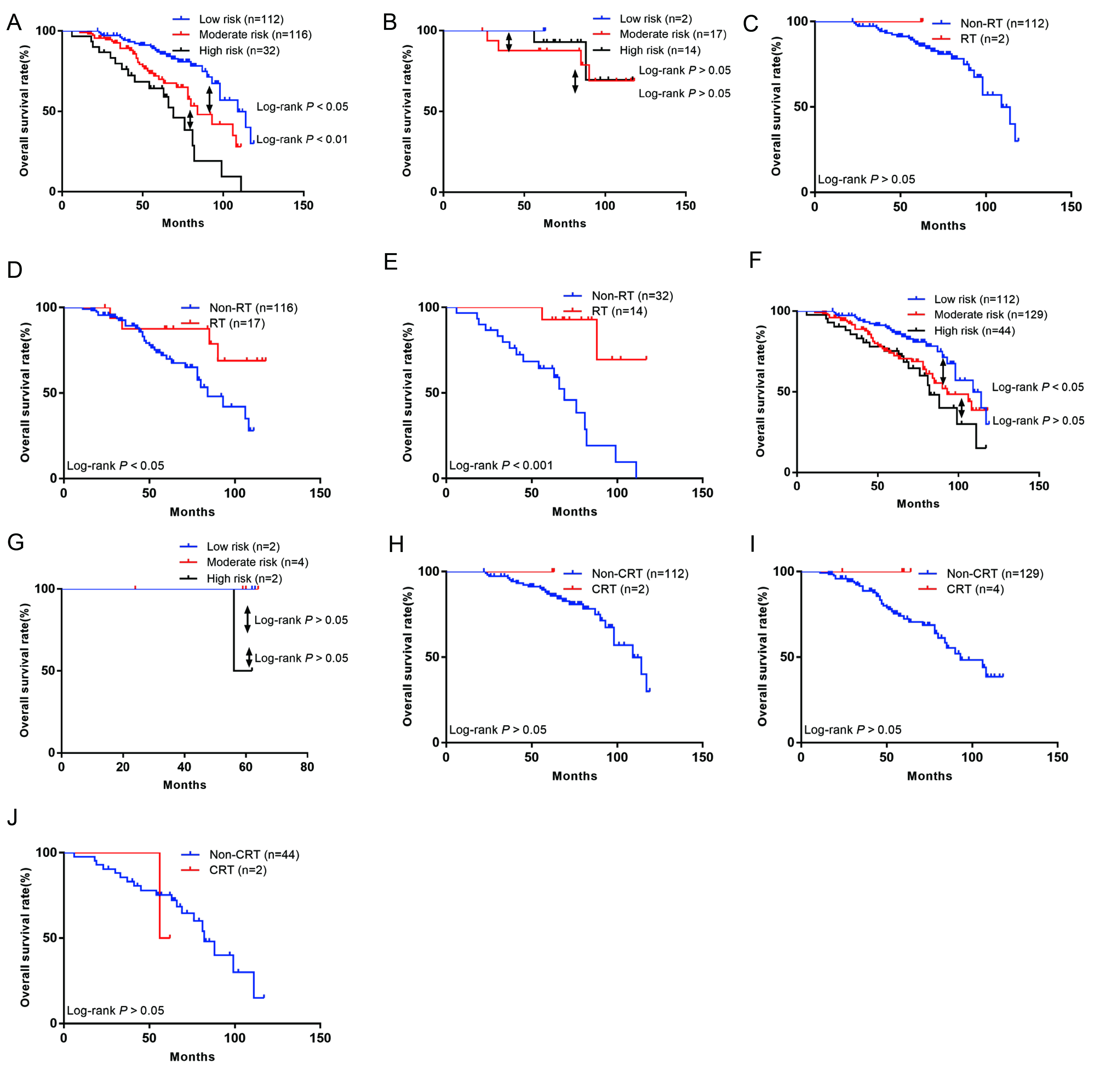

Supplement: oyae068_suppl_Supplementary_Figures_S1-S3 [file oyae068_suppl_supplementary_figures_s1-s3.zip › figure S3.tif]

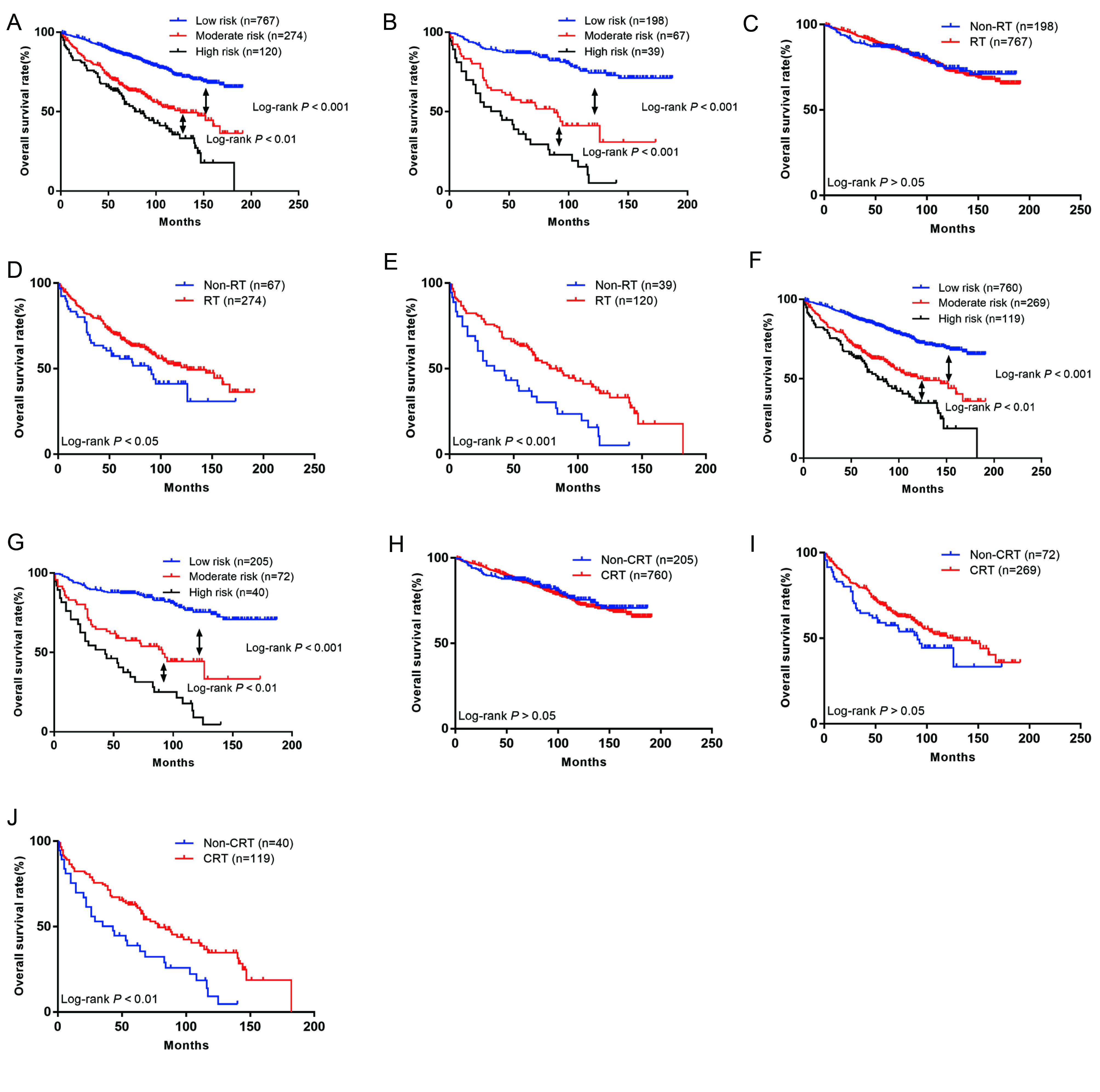

Supplement: oyae068_suppl_Supplementary_Figures_S1-S3 [file oyae068_suppl_supplementary_figures_s1-s3.zip › figure S1.tif]

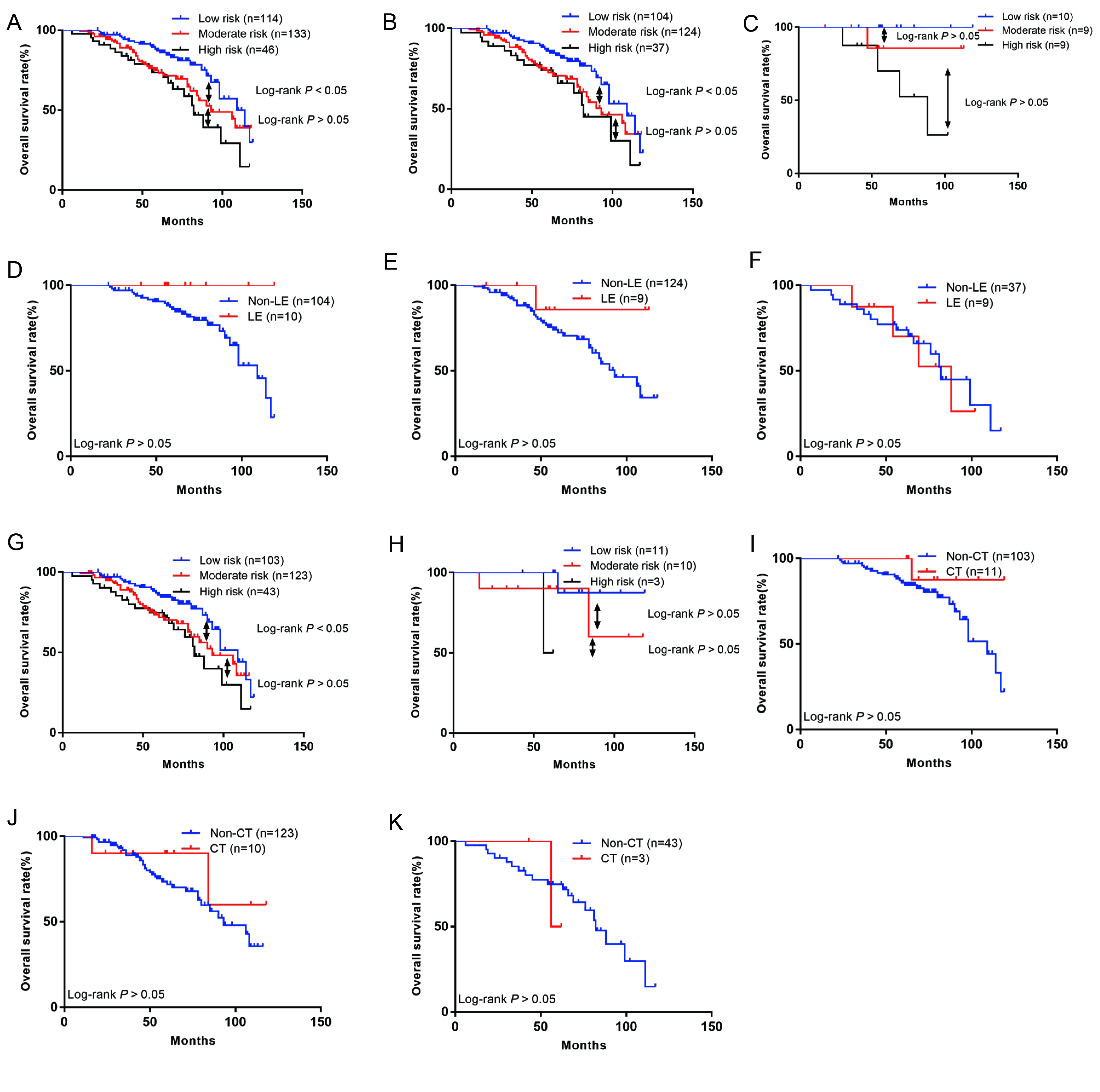

Supplement: oyae068_suppl_Supplementary_Figures_S1-S3 [file oyae068_suppl_supplementary_figures_s1-s3.zip › figure S2.tif]
